# Supplementary material for: Personalized cancer therapy prioritization based on driver alteration co-occurrence patterns
Source: Genome Med. 2020 Sep 9;12:78. doi: 10.1186/s13073-020-00774-x (PMC7488324; doi:10.1186/s13073-020-00774-x)
Supplement: Supplementary file 1 — Additional file 1. This compressed folder contains all the supplementary tables referenced throughout the manuscript, together with their description. [file 13073_2020_774_MOESM1_ESM.zip › SupplementaryTables/Table Descriptions.docx]

**Supplementary Table S1.** *TCT4U drug family annotation.* Mapping of TCT4U drugs to drug families and drugs for which we could identify at least one approved or experimental biomarker of response reported in the Cancer bioMarkers database accessed in the 25^th^ of August 2017.

**Supplementary Table S2.** This dataset contains the driver mutations, copy number alterations and biomarkers identified in the NIBR PDX Encyclopedia (NBIR PDXE) according to the Cancer Genome Interpreter. It also contains the whole collection of TCT4U DCO networks. The *DCO Nodes Table* contains the information about each individual gene in each DCO network it participates in. We provide information about the specific treatment arm from which the DCO network was inferred (drug), the number of responder and non-responder PDXs with and without driver alterations in it (mut_sens, mut_res, no_mut_sens, and no_mut_res), the estimated probability that the alteration rate is higher in responder than in non-responders (DiffD), the average feature importance of each driver across all folds in the LOOCV (CatBoost_Importance), and the average SHAP value of each gene in PDXs with and without driver alterations in it. Moreover, we provide the chromosomal location of each gene and we indicate whether it is covered by MSK-IMPACT and FoundationMedicine targeted gene panels. The *DCO Edges Table* describes the pairs of drivers (gene A – gene B) that appear co-altered more often than expected in each of the three DCO networks inferred from a treatment arm (drug) that contributed to the prediction of response in the LOOCV. We provide information about the number of responder and non-responder PDXs with and without a given pair of co-occuring driver alterations in (coocurr_Resp, coocurr_NonResp, no_cooccurr_Resp, no_cooccurr_NonResp), the observed co-alteration rate (Ps), and the estimated probability that the observed and expected co-alteration rate under the null model are different (Ps_proba). Moreover, we also provide de probability that a given pair co-occurs more often in responder than in non-responder PDXs (Ps_diff_proba) and the proportion of cases in which this actual probability is larger than the observed in the 1,000 random permutations (Ps_diff_eval). We also indicate whether a given pair co-occurs within the same chromosome and/or cytogenetic band and the distance in megabases (Mb) between its constituent genes. Finally, we also report the strength of the interaction as determined by CatBoost (CatBoost_interaction) and we report the effect of having a driver alteration in gene A on the SHAP value of gene B, and viceversa.

**Supplementary Table S3**. *Quantification of the overlap between DCO networks and previously reported biomarkers.* For each drug, we provide the quantification of the overlap between DCO networks and experimental or approved biomarkers. We also quantified the overlap of the aggregated counts.

**Supplementary Table S4**. We provide the predictive performance of all models in terms of the balanced accuracy calculated separately for each treatment arm. We also provide their average across treatments.

**Supplementary Table S5.** Summary of the experimental validation of 14 high confidence TCT4U drug response predictions. For each drug-PDX pair we report the anticipated response according to approved or experimental biomarkers (bioMarkers db) or by TCT4U. We also provide the SHAP values attributed to the driver alterations explaining TCT4U predictions. Altogether, we provide the experimentally determined response. Drug-PDX pairs were classified following the mRECIST criteria into responders (SD, PR or CR) and non-responders (PD). Additionally, we report the time at which the Best Response was observed and the tumor growth achieved at this time point with respect to the baseline, both in the treatment and placebo arms. We also provide the tumor growth inhibition in the treatment arm with respect to the placebo.

**Supplementary Table S6**. *Clinical and molecular profile (Mutations and CNAs) of a cohort of 216 advanced metastatic breast cancer patients treated with a CDK4/6 inhibitor in combination with an aromatase inhibitor at the Memorial Sloan Kettering Cancer Center (MSKCC).* We report the somatic mutations and copy number alterations detected in pre-treatment biopsies of the tumors using MSK-IMPACT gene panel. Genomic data has been annotated using MSKCC knowledge base database (OncoKb [13]) and also with clinical data indicating the line of therapy (txline), the time to progression in months (pfsm), and the outcome variable (pfs_event), which takes a value of 1 when the treatment was discontinued because of tumor progression or a value of 0 for other causes of discontinuation.
