# Supplementary material for: Personalized cancer therapy prioritization based on driver alteration co-occurrence patterns
Source: Genome Med. 2020 Sep 9;12:78. doi: 10.1186/s13073-020-00774-x (PMC7488324; doi:10.1186/s13073-020-00774-x)
Supplement: Supplementary file 3 — Additional file 3. This pdf file contains the Supplementary Figures that are referenced throughout the manuscript, together with their figure legends. Higher resolution figures and extended data are available upon reasonable request. [file 13073_2020_774_MOESM3_ESM.pdf]

## *Supplementary Figures*

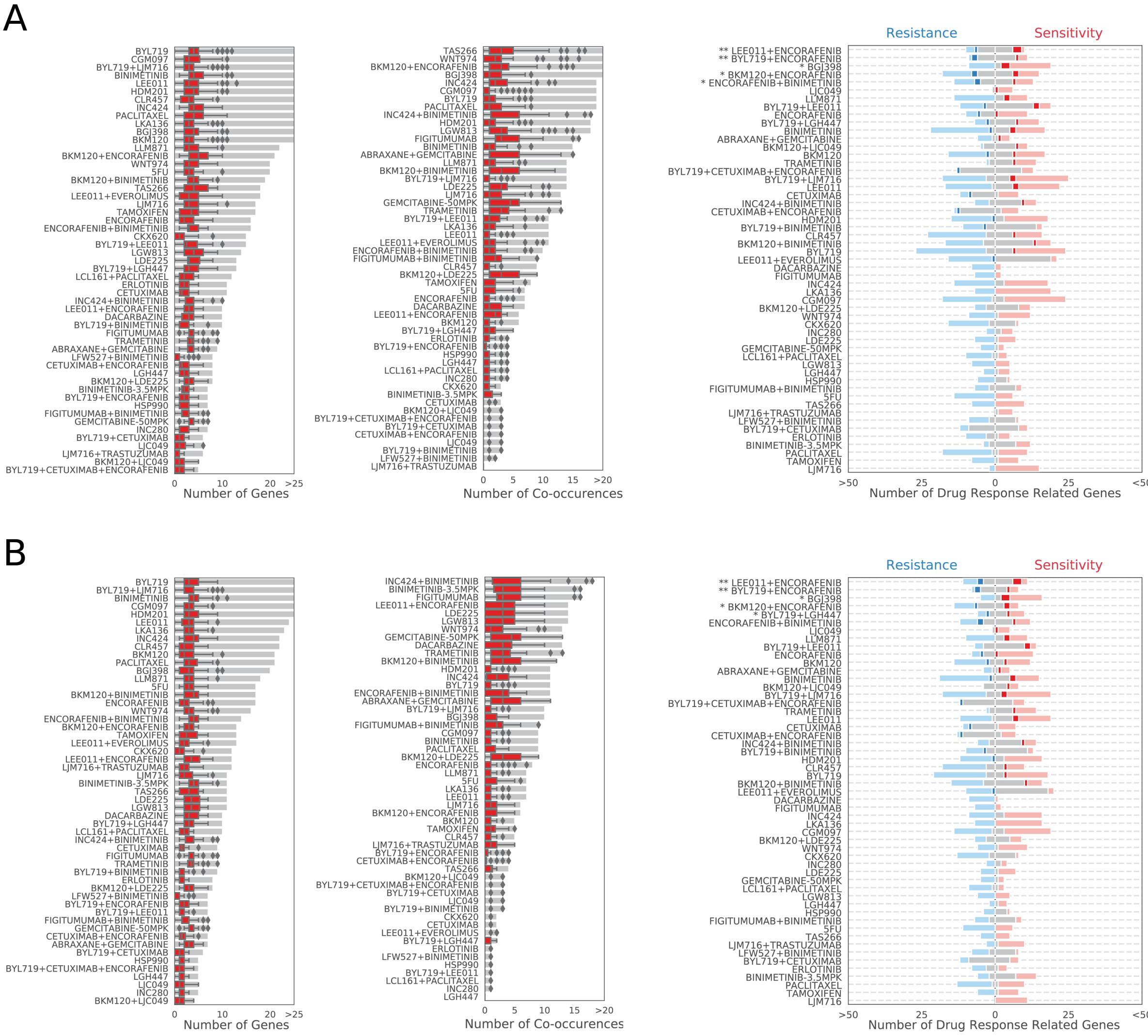


**Figure S1.** *Characterization of the DCO networks derived from targeted gene panels*. **(A)** DCO networks derived from MSK-IMPACT gene panel have 5 to 42 driver genes (median of 13, IQR: 8-21) and up to 21 pairs of drivers (median of 9, IQR: 4-15). Each PDX has a median of 3 altered drivers (IQR: 2-4) and 1 driver alteration co-occurrence (IQR: 0-2). **(B)** DCO networks derived from Foundation Medicine gene panel have 4 to 33 driver genes (median of 11, IQR: 7-17) and up to 18 pairs of drivers (median of 7, IQR: 3-11). Each PDX has a median of 0 altered drivers (IQR: 0-2) and 2 driver alteration co-occurrences (IQR: 0-6).

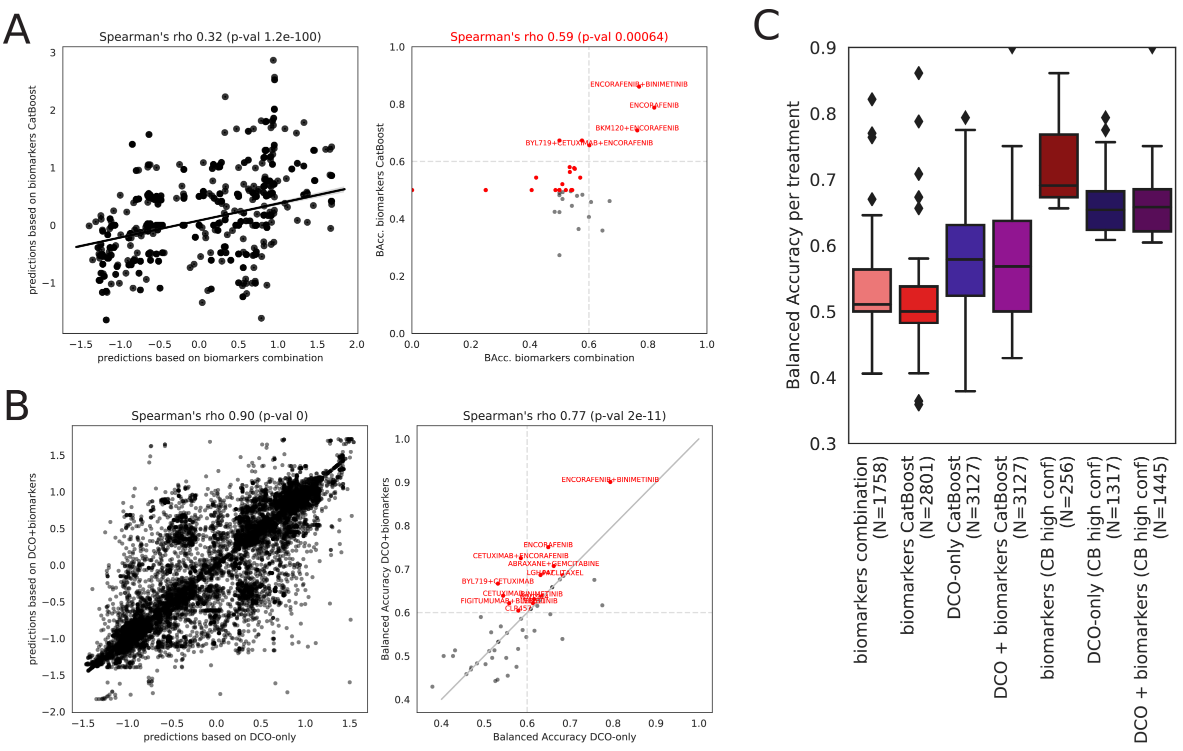


**Figure S2. (A)** Comparison of the predictive performance of the simple combination of known biomarkers to that of the predictions generated with CatBoost classifiers trained on the same features, and integrated using TCT4U pipeline. We observed a significant correlation when treatment responses were predictable by either of the two approaches with a balanced accuracy of 0.5 or more. **(B)** Comparison of the predictive performance of an integrated model trained on both DCO and previously reported biomarkers to that of DCO-only based models. Considering both sets of features modestly improved the performance of 20 treatment models, increasing the number of treatments attaining a balanced accuracy of 0.6 from 21 to 23 and the total number of high-confidence predictions from 1,317 to 1,445. (**C**) Summary boxplot showing the predictive performance of all models.

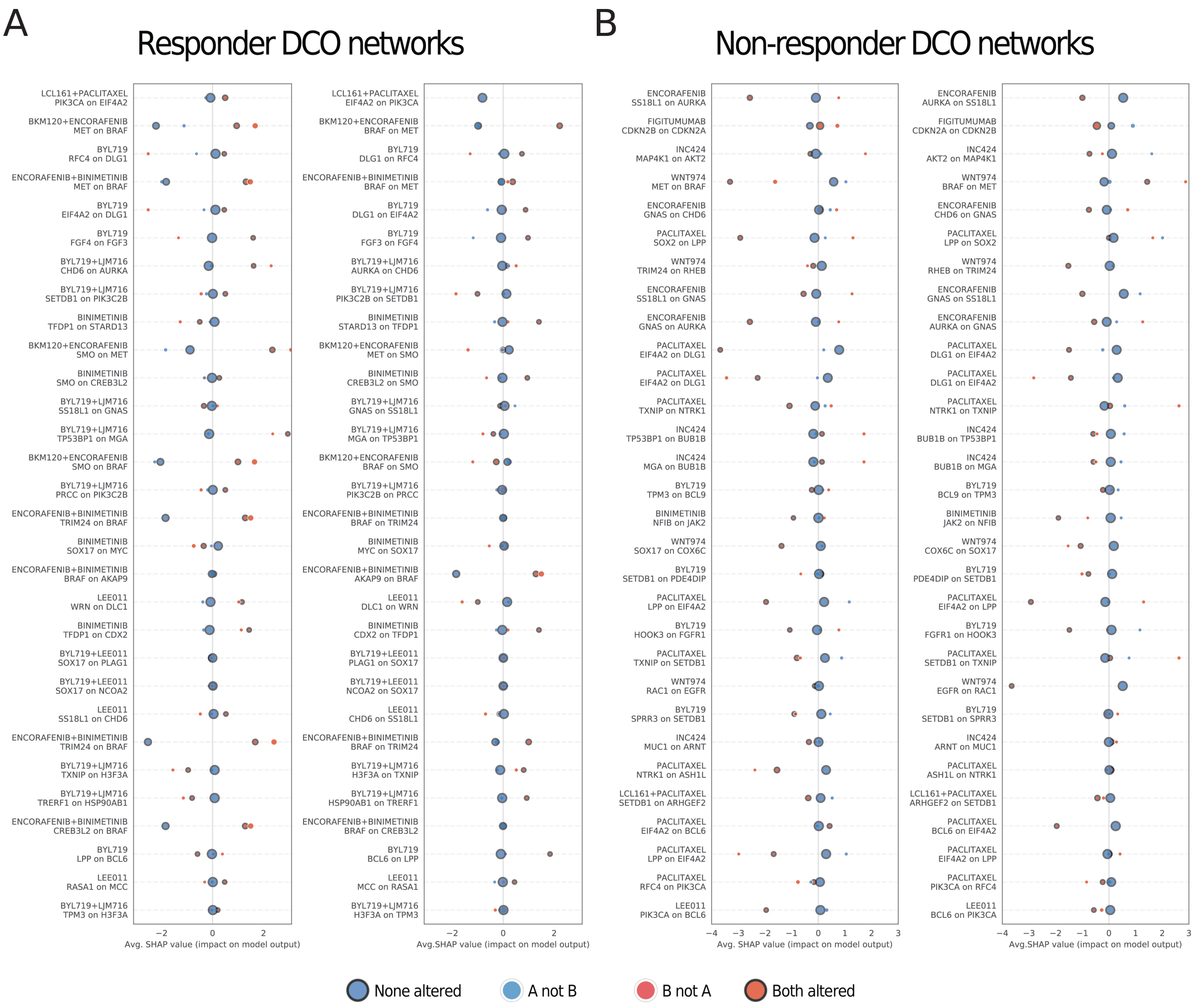


**Figure S3.** SHAP interaction plots of co-occurring pairs of drivers located in the same chromosomal arm that might be genomically linked. Each row shows the effect of having a driver alteration in gene A on the distribution of SHAP values of gene B. Each point represents the average SHAP value of PDXs classified on the basis of the status of the two drivers that tend to be co-altered. The size of the points is proportional to the number of PDXs that belong to each of the four resulting categories. Beyond the top 30 strongest interactions shown in this figure, we provide the full set of interactions and their impact on SHAP values in Table S1.
